# Evaluation of Polytetrafluoroethylene (PTFE) Tape for Gingival Displacement Using CAD/CAM Analysis and Scanning Electron Microscopy (SEM)

**DOI:** 10.3390/jfb17080408

**Published:** 2026-08-17

**Authors:** Laura Monica Rusu, Antonio Pop, Stanca Cuc, Mircea Aurelian Rusu, Sorina Sava, Anca Labunet, Cristina Iosif, Adriana Objelean

**Affiliations:** 1 Dental Materials Discipline, Department of Prosthodontics and Dental Materials, Faculty of Dental Medicine, “Iuliu Hațieganu” University of Medicine and Pharmacy, 400012 Cluj-Napoca, Romania; dascalu.monica@umfcluj.ro (L.M.R.); pop.antonio@elearn.umfcluj.ro (A.P.); savasorina@elearn.umfcluj.ro (S.S.); jiglau.anca@umfcluj.ro (A.L.); mecicovschi.cristina@umfcluj.ro (C.I.); adriana.caracostea@elearn.umfcluj.ro (A.O.); 2 “Raluca Ripan” Institute of Research in Chemistry, “Babeș Bolyai” University, 400294 Cluj-Napoca, Romania; stanca.boboia@ubbcluj.ro; 3 Department of Manufacturing Engineering, Machine Building, Technical University of Cluj-Napoca, 400641 Cluj-Napoca, Romania

**Keywords:** gingival displacement, polytetrafluoroethylene tape, PTFE, gingival retraction cord, Teflon tape

## Abstract

**Background:** This in vitro study compared the effectiveness of polytetrafluoroethylene (PTFE) tape and a conventional cotton retraction cord for gingival displacement and evaluated the mechanical and microstructural characteristics of PTFE tape. **Methods:** Eight standardized prepared teeth with artificial gingiva were evaluated under three conditions: control, cotton retraction cord, and PTFE tape. After each condition, impressions were digitized using a CAD/CAM system. Sulcular width was measured at four predefined locations and averaged for statistical analysis. PTFE tape was also evaluated by tensile testing before and after sterilization and by scanning electron microscopy (SEM). **Results:** Mean sulcular widths were 0.716 ± 0.118 mm (control), 0.851 ± 0.140 mm (cotton cord), and 0.907 ± 0.099 mm (PTFE). Repeated-measures ANOVA showed a significant effect of the displacement method (*p* < 0.001), with PTFE producing significantly greater sulcular enlargement than cotton cord. Qualitative SEM analysis revealed a more homogeneous impression surface morphology following PTFE removal. Sterilization did not significantly affect the tensile properties or microstructural integrity of PTFE tape. **Conclusions:** Under standardized in vitro conditions, PTFE tape produced the highest gingival sulcular enlargement and maintained favorable mechanical and microstructural properties, supporting its potential as an alternative gingival displacement material. Further clinical studies are required to confirm these findings.

## 1. Introduction

Clinical performance and long-term success of indirect dental restorations are closely related to the accurate execution of multiple clinical procedures. Among these, the management of gingival tissues and the preservation of gingival esthetics represent particularly demanding steps. The primary objective of gingival tissue management is to maintain the physiological appearance of healthy gingiva, which requires optimal periodontal conditions before treatment and minimal tissue trauma during clinical procedures. Preservation of gingival health is best achieved by reducing mechanical and chemical irritation and by preventing direct contact between restorative materials and the marginal gingiva. Favorable gingival architecture plays a crucial role in the overall esthetic outcome of fixed prosthodontic restorations, and it is most predictably obtained when preoperative gingival health is ensured and intraoperative trauma is carefully controlled [1,2,3,4,5,6,7].

Inadequate adaptation of restorations with subgingival margins has been associated with a range of biological complications, including increased biofilm retention, development of secondary carious lesions, and inflammatory changes in the surrounding periodontal tissues. Consequently, meticulous management of gingival displacement and impression-taking procedures is essential to ensure optimal marginal accuracy and to reduce the risk of adverse periodontal outcomes [8,9,10,11].

Gingival displacement (GD) is a fundamental procedure in fixed prosthodontics aimed at temporarily displacing the marginal gingival tissues away from the tooth surface to facilitate the accurate recording of subgingival finish lines. By creating both horizontal and vertical sulcular enlargement, GD improves access to preparation margins and provides sufficient space for impression material penetration, thereby enhancing the accuracy of the definitive impression. Previous studies have suggested that a sulcular width of approximately 0.2 mm is necessary to allow adequate flow of impression materials and to minimize the risk of tearing or distortion during impression removal. In addition to improving marginal reproduction, gingival displacement contributes to moisture control and hemostasis, both of which are essential for predictable impression making and adhesive restorative procedures [2,3,12].

Various gingival displacement techniques have been described, including mechanical, chemical, surgical, and combined approaches. Among these, mechanical displacement using gingival retraction cords remains one of the most widely adopted techniques because of its simplicity, effectiveness, and clinical predictability. Retraction cords may be used alone or in combination with hemostatic agents to improve bleeding control during impression procedures [2,13,14,15].

Despite their widespread clinical acceptance, conventional cotton retraction cords present several limitations. Excessive packing forces or improper placement may result in gingival trauma, postoperative discomfort, inflammation, or gingival recession. Furthermore, residual hemostatic agents associated with medicated cords may adversely affect the interaction between the prepared tooth surface and impression materials. These limitations, together with the increasing demand for minimally traumatic clinical procedures, have stimulated interest in alternative gingival displacement materials and techniques [1,13,16,17,18].

Among the alternative materials proposed for gingival displacement, polytetrafluoroethylene (PTFE) tape has attracted increasing interest in restorative and prosthetic dentistry. Commonly known as Teflon tape, PTFE has been extensively used in a variety of clinical applications, including the protection of implant screw-access channels, isolation during adhesive procedures, management of undercuts during impression making, and prevention of excess cement extrusion into the sulcus [19,20,21,22,23,24,25]. The growing adoption of PTFE in these procedures reflects its favorable handling characteristics and biocompatibility. The increasing interest in PTFE tape is largely related to its unique physicochemical properties. PTFE is chemically inert, hydrophobic, dimensionally stable, resistant to moisture absorption, and capable of withstanding conventional sterilization procedures without significant degradation. Furthermore, its non-fibrous and non-adhesive surface facilitates insertion and removal while reducing the likelihood of interaction with surrounding restorative materials. These characteristics suggest that PTFE tape may represent a minimally traumatic alternative to conventional cotton retraction cords for temporary gingival displacement [26,27,28,29,30,31,32].

Although PTFE tape has been increasingly adopted in restorative and implant dentistry because of its favorable handling characteristics and physicochemical properties, evidence supporting its use as a gingival displacement material remains limited. The available studies have primarily focused on clinical performance and short-term outcomes, while comprehensive investigations comparing PTFE tape with conventional cotton retraction cords are still scarce. In particular, limited information is available regarding the capacity of PTFE tape to achieve clinically relevant sulcular enlargement, its mechanical behavior under tensile loading, the potential effects of sterilization on its microstructure, and its interaction with impression materials. As a result, the selection of gingival displacement materials is often based on clinical preference rather than on standardized experimental evidence. Further research is therefore required to establish whether PTFE tape represents a predictable and clinically effective alternative to conventional cotton retraction cords [26,27,33,34].

Therefore, the present in vitro study was designed to compare conventional cotton retraction cord and PTFE tape as gingival displacement materials. A standardized three-dimensional printed dental model with an artificial gingival tissue was used to evaluate the displacement effectiveness of both materials. Sulcular enlargement was quantitatively assessed through CAD/CAM digital analysis. In addition, the mechanical behavior of PTFE tape was characterized through tensile testing, while its microstructural features before and after sterilization were examined using scanning electron microscopy (SEM). Furthermore, the interaction between the gingival displacement materials and the impression material was qualitatively investigated by SEM analysis of specimens obtained after impression making and removal of the retraction material.

The primary objective of this study was to compare the ability of cotton retraction cord and PTFE tape to achieve relevant gingival sulcus enlargement under standardized in vitro conditions. Secondary objectives included evaluating the mechanical properties of PTFE tape, assessing possible microstructural alterations induced by sterilization, and investigating surface interactions between the displacement materials and the impression material. The null hypothesis was that no significant differences would be observed between the tested gingival displacement materials regarding sulcular enlargement and that no qualitative differences would be identified in their interaction with the impression material under the experimental conditions of the present study.

## 2. Materials and Methods

### 2.1. 3D-Printed Models

**Figure 1:**
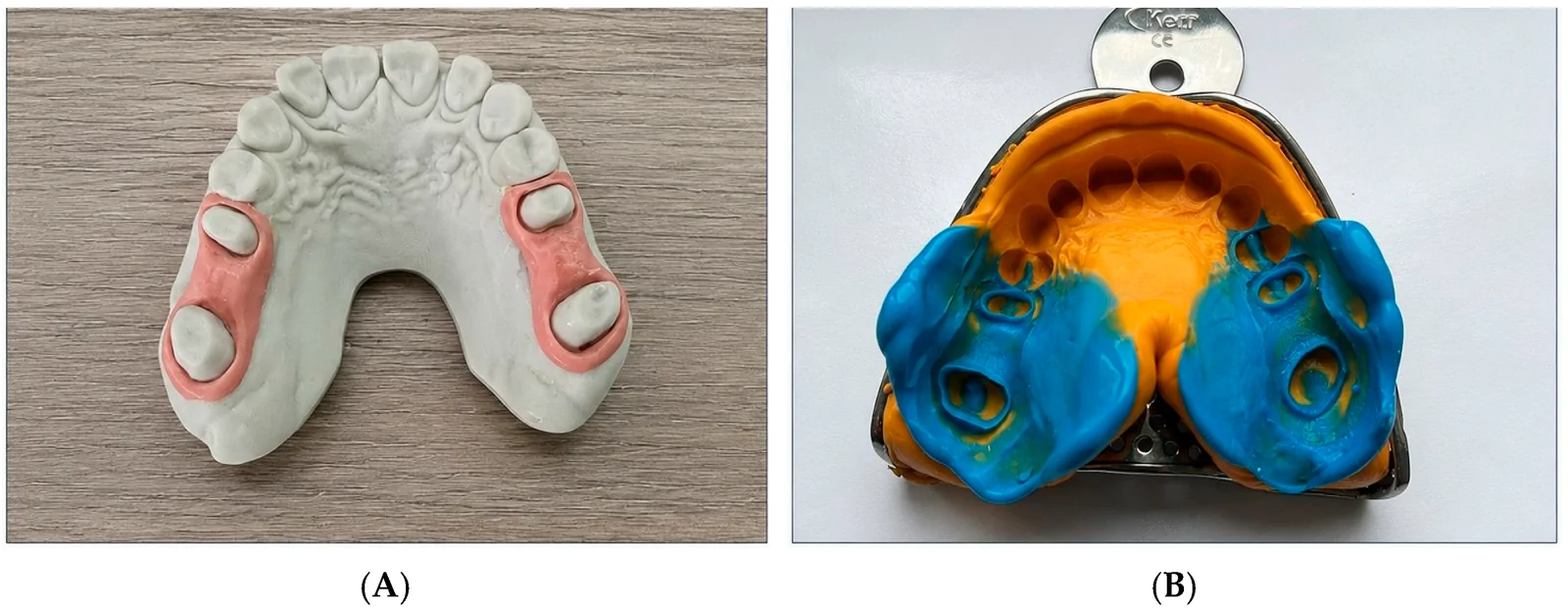
Experimental 3D-printed model and impression: (**A**) The study was performed on a 3D-printed model with prepared teeth and artificial gingiva simulating periodontal tissues. (**B**) An impression was taken after gingival displacement using the tested materials.

Standardized dental models were designed using computer-aided design (CAD-CAM) (Ceramill Map 200, Amann Girrbach, Koblach, Austria) software (version 4.8) and fabricated through additive manufacturing techniques (Figure 1A). Each model incorporated tooth preparation with a circumferential subgingival finish line, ensuring uniform marginal configuration across all specimens. Artificial gingival tissues were reproduced using a silicone elastomer material (Gingifast Rigid, Zhermack, Badia Polesine, Italy) to simulate gingival tissues during displacement procedures.

The artificial gingival model was selected to provide standardized and reproducible experimental conditions for comparing the tested gingival displacement materials. The purpose of the model was not to reproduce the exact physiological dimensions or biomechanical behavior of the natural gingival sulcus, but to enable direct comparison of the evaluated displacement techniques under identical laboratory conditions. All specimens were fabricated using the same silicone material and preparation protocol to minimize inter-specimen variability.

Standardization of the digital design, manufacturing process, and material composition ensured consistent specimen geometry and minimized experimental variability among all tested models.

### 2.2. Gingival Displacement Procedures

The experimental unit was the prepared tooth. Each of the eight prepared teeth was evaluated under three experimental conditions according to the gingival displacement protocol employed. First, an impression was obtained without gingival displacement (Control condition A, *n* = 8), serving as the reference for sulcular measurements. Subsequently, gingival displacement was performed using a non-impregnated hydrophilic cotton retraction cord (Retraflex Retraction Cord, Size 2, Biodinamica, Brazil) (Condition B, *n* = 8), followed by impression making. Finally, gingival displacement was performed using a commercially available polytetrafluoroethylene (PTFE) dental tape (Remer 572, Remer, China; 12.7 mm width, 0.075 mm thickness) (Condition C, *n* = 8), and a third impression was obtained. The PTFE tape is a biologically inert material intended for dental applications, including isolation of adjacent teeth, protection of prosthetic preparations, endodontic procedures, and use as a barrier during impression procedures. Thus, each prepared tooth served as its own control, minimizing inter-specimen variability throughout the experimental procedure.

To ensure dimensional standardization between the two displacement materials, the PTFE tape was adapted to approximate the dimensions of the conventional cotton retraction cord. Briefly, an impression of the retraction cord was obtained using an addition silicone impression material, and the resulting negative mold was used as a guide for shaping and adapting the PTFE tape to achieve a comparable cross-sectional configuration before insertion into the artificial gingival sulcus.

All gingival displacement and impression-making procedures were performed by a single experienced operator under standardized laboratory conditions. Both the cotton retraction cord and the PTFE tape were inserted using the same gingival cord packing instrument (PRIMA gingival retraction instrument with two rounded 1.5 mm working ends, stainless steel) and an identical insertion technique. Prior to insertion, the PTFE tape was gently twisted to facilitate handling and adaptation within the artificial gingival sulcus. The retraction materials remained in position for 5 min before removal. This exposure time was selected to simulate routine clinical practice and to ensure consistent testing conditions among all experimental conditions. Although the insertion technique and packing instrument were standardized, the insertion force was not quantitatively measured.

### 2.3. Assessment of Gingival Sulcus Enlargement

Following gingival displacement, definitive impressions were obtained using a sandwich technique with addition silicone impression materials (Elite HD+, Zhermack, Italy), combining putty and light-body consistencies to ensure accurate reproduction of the preparation margins and surrounding gingival structures (Figure 1B). After complete setting, the impressions were scanned using an optical CAD/CAM scanner (Ceramill Map 200, Amann Girrbach, Koblach, Austria), software version 4.8, and three-dimensional digital models were generated for subsequent analysis. According to the manufacturer’s specifications, the Ceramill Map 200 scanner provides a scanning accuracy of 6 μm for conventional prosthodontic applications and employs blue-light structured-light technology with high-definition adaptive scanning to capture fine geometric details.

**Figure 2:**
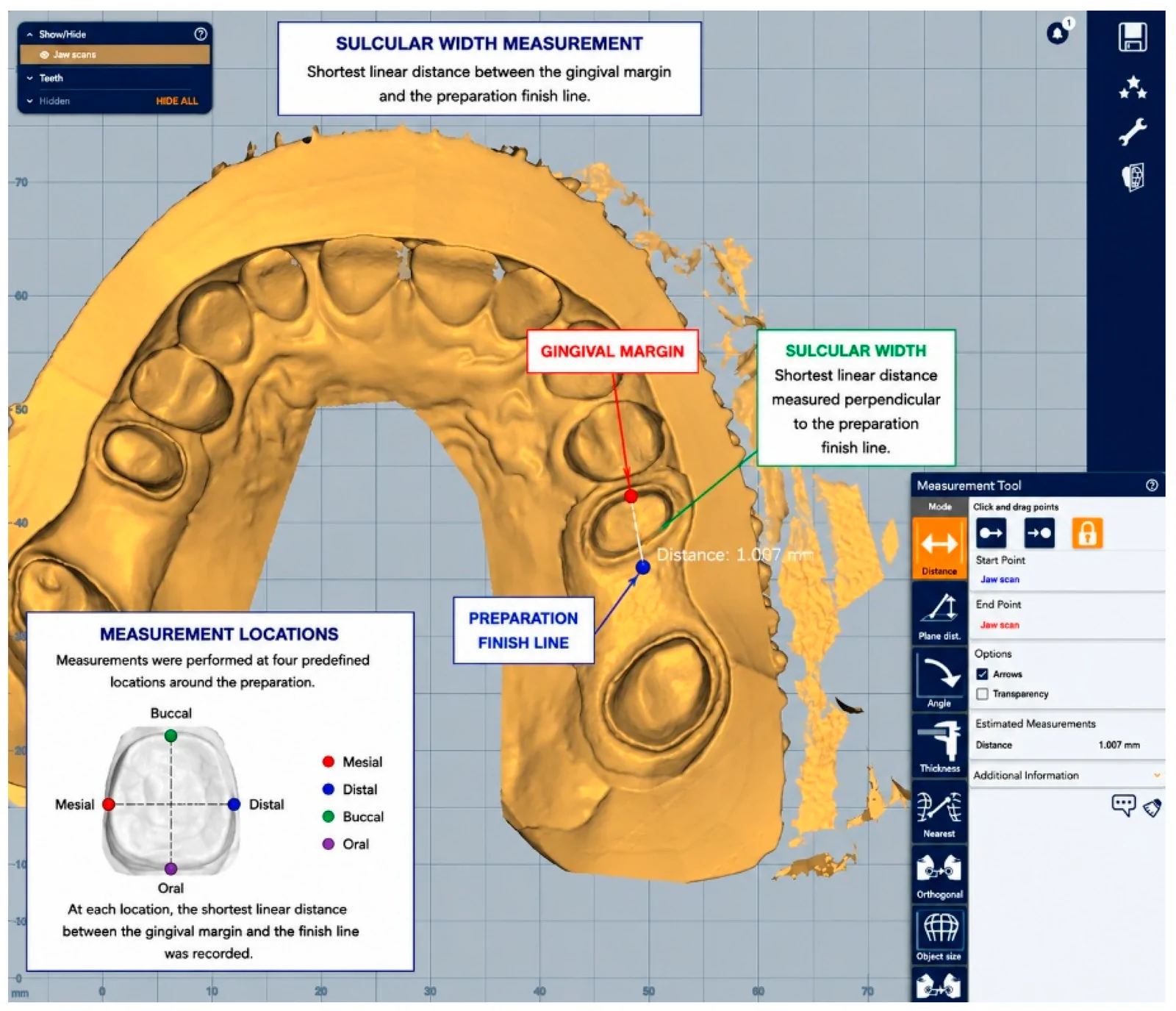
Measurement of sulcular width on digital models. Sulcular width was defined as the shortest perpendicular linear distance between the gingival margin reproduced in the impression and the preparation finish line.

The extent of gingival sulcus enlargement produced by each displacement material was evaluated on the digital models generated from the scanned impressions. Sulcular width was defined as the shortest perpendicular linear distance between the gingival margin reproduced in the impression and the preparation finish line. Measurements were performed perpendicular to the preparation finish line at four standardized locations around each prepared tooth (mesial, distal, buccal, oral) to provide a comprehensive circumferential assessment of gingival displacement (Figure 2). The recorded distance represented the horizontal opening of the gingival sulcus available for impression material penetration rather than vertical exposure of the preparation margin. For each tooth and experimental condition, the four site-specific measurements were averaged to obtain a single representative sulcular width value for statistical analysis.

All measurements were carried out using the Ceramill CAD/CAM software associated with the scanning system (Ceramill Map 200, Amann Girrbach, Austria). For each measurement site, the preparation finish line served as the reference line, and the corresponding reproduced gingival margin was identified as the second reference landmark. The shortest perpendicular distance between these landmarks was then recorded.

All scanning procedures and digital measurements were performed by a single examiner under identical evaluation conditions using a standardized measurement protocol. Prior to the study, the examiner underwent a calibration procedure by repeating measurements on randomly selected sites after a seven-day interval using the same measurement protocol. Intra-examiner reproducibility was assessed using the intraclass correlation coefficient (ICC), demonstrating excellent agreement (ICC = 0.999).

### 2.4. Mechanical Testing of PTFE Tape

To evaluate the mechanical behavior of the polytetrafluoroethylene tape used for gingival displacement, tensile testing was performed under controlled laboratory conditions. The PTFE material was investigated in four different conditions: non-sterilized tape, sterilized tape, twisted tape, and sterilized twisted tape. The twisted configuration was included to simulate the clinical manipulation of PTFE tape prior to its insertion into the gingival sulcus, as twisting facilitates handling, adaptation, and condensation of the material during gingival displacement procedures.

Mechanical testing was conducted using a universal testing machine (Lloyd Instruments, AMETEK Inc., West Sussex, UK) equipped with a 5 kN load cell. All specimens were subjected to uniaxial tensile loading until failure, with a maximum applied load of 100 N. Before testing, the PTFE tape was cut into standardized 50 mm long specimens to allow secure fixation in the testing machine grips and to ensure reproducible tensile testing conditions. The specimens were secured to ensure axial loading and prevent slippage throughout the test.

For each experimental condition, eight specimens were evaluated in every group. The ultimate tensile strength (MPa) and maximum load at failure (N) were recorded for each specimen, and the results were expressed as mean ± standard deviation.

### 2.5. Scanning Electron Microscopy (SEM) Analysis

#### 2.5.1. SEM Analysis of PTFE Tape

The microstructural characteristics of the polytetrafluoroethylene tape were investigated by scanning electron microscopy to evaluate the potential effects of sterilization on surface morphology. Two groups were analyzed: PTFE tape as supplied by the manufacturer and PTFE tape subjected to conventional steam sterilization procedures routinely used in dental practice.

Before SEM examination, the specimens were cut into standardized segments and mounted on aluminum stubs using conductive carbon adhesive tabs. Observations were performed using a scanning electron microscope (Inspect S, FEI, Eindhoven, The Netherlands) under vacuum conditions. Micrographs were acquired at magnifications ranging from ×100 to ×5000 in order to evaluate both the overall architecture and the fine surface features of the material. Particular attention was given to the identification of morphological alterations potentially induced by sterilization, including changes in surface texture, fibrillation patterns, microcracks, irregularities, and structural discontinuities. All images were recorded using identical operating parameters to allow direct comparison between sterilized and non-sterilized specimens.

#### 2.5.2. SEM Assessment of Material–Impression Interactions

In addition, the interaction between the gingival displacement materials and the impression material was assessed by SEM. One cotton retraction cord specimen and one PTFE tape specimen were embedded separately into an addition silicone impression material under standardized conditions. Following complete polymerization, the displacement materials were removed, and the corresponding silicone specimens were prepared for SEM examination. The analysis focused on the morphology of the impression material at the material–impression interface, with special emphasis on residual surface imprints, material adhesion, void formation, and other microstructural features that could influence the accuracy of impression reproduction. SEM observations were performed at magnifications ranging from ×50 to ×2000 using the same microscope and acquisition parameters.

### 2.6. Statistical Analysis

All quantitative data were expressed as mean ± standard deviation (SD). Ninety-five percent confidence intervals (95% CI) were calculated for the mean sulcular width values. For each tooth and experimental condition, the four site-specific sulcular width measurements (mesial, distal, buccal, oral) were averaged to obtain a single representative value for statistical analysis. Normality of the data distribution was assessed using the Shapiro–Wilk test. Because each prepared tooth was evaluated under all three experimental conditions (control, cotton retraction cord, and PTFE tape), differences in gingival sulcus enlargement were analyzed using one-way repeated-measures analysis of variance (ANOVA). When a significant overall effect was detected, pairwise comparisons were performed using the Bonferroni correction. Effect size was expressed as partial eta squared (η^2^). Tensile strength and maximum load data obtained from the mechanical characterization of PTFE specimens were analyzed using one-way ANOVA. Statistical significance was established at *p* < 0.05.

## 3. Results

### 3.1. Digital Assessment of Sulcular Enlargement

The effectiveness of the gingival displacement procedures was assessed using dimensional analysis of digital models generated from scanned impressions. Sulcular width was measured at four predefined locations (mesial, distal, buccal, oral) around each prepared tooth. For each tooth and experimental condition, the four site-specific measurements were averaged to obtain a single representative sulcular width value.

**Figure 3:**
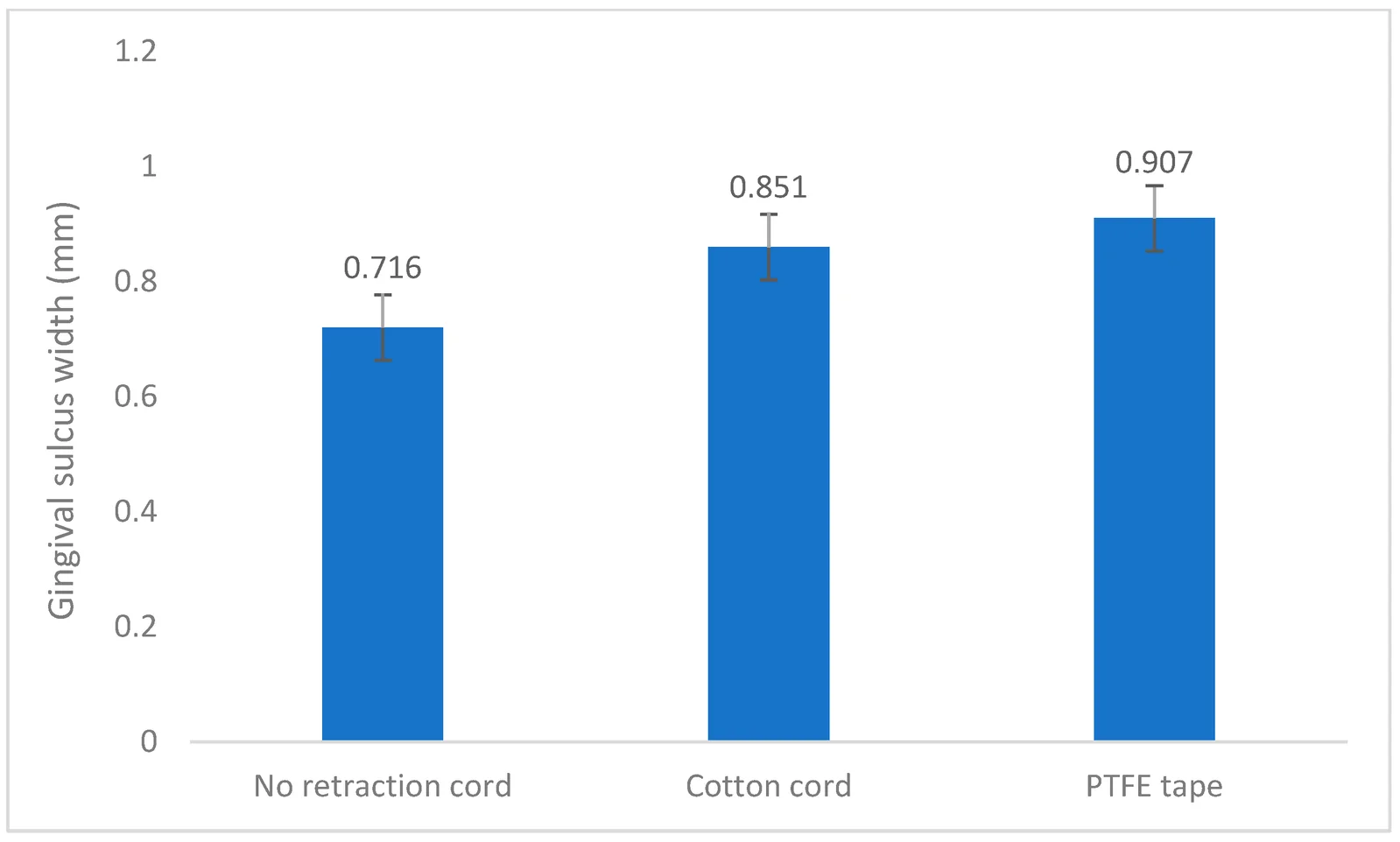
Mean gingival sulcus width measured under three experimental conditions. Data are presented as mean ± SD (*n* = 8). Each value represents the mean of four standardized measurement sites per tooth. Error bars represent the standard deviation.

The control condition exhibited a mean sulcular width of 0.716 ± 0.118 mm (95% CI: 0.617–0.815 mm). The conventional cotton retraction cord increased the mean sulcular width to 0.851 ± 0.140 mm (95% CI: 0.733–0.968 mm), whereas PTFE tape produced the greatest mean sulcular width, reaching 0.907 ± 0.099 mm (95% CI: 0.824–0.990 mm) (Figure 3).

One-way repeated-measures ANOVA demonstrated a statistically significant effect of the gingival displacement condition on sulcular width (F(2,14) = 136.87, *p* < 0.001). Bonferroni-adjusted pairwise comparisons showed significantly greater sulcular enlargement following both cotton retraction cord and PTFE tape compared with the control condition (both *p* < 0.001). In addition, PTFE tape produced significantly higher sulcular enlargement than the conventional cotton retraction cord (*p* = 0.024).

Compared with the control condition, the cotton retraction cord increased the mean sulcular width by approximately 18.9%, whereas PTFE tape increased it by approximately 26.7%.

### 3.2. Mechanical Testing of PTFE Tape

**Figure 4:**
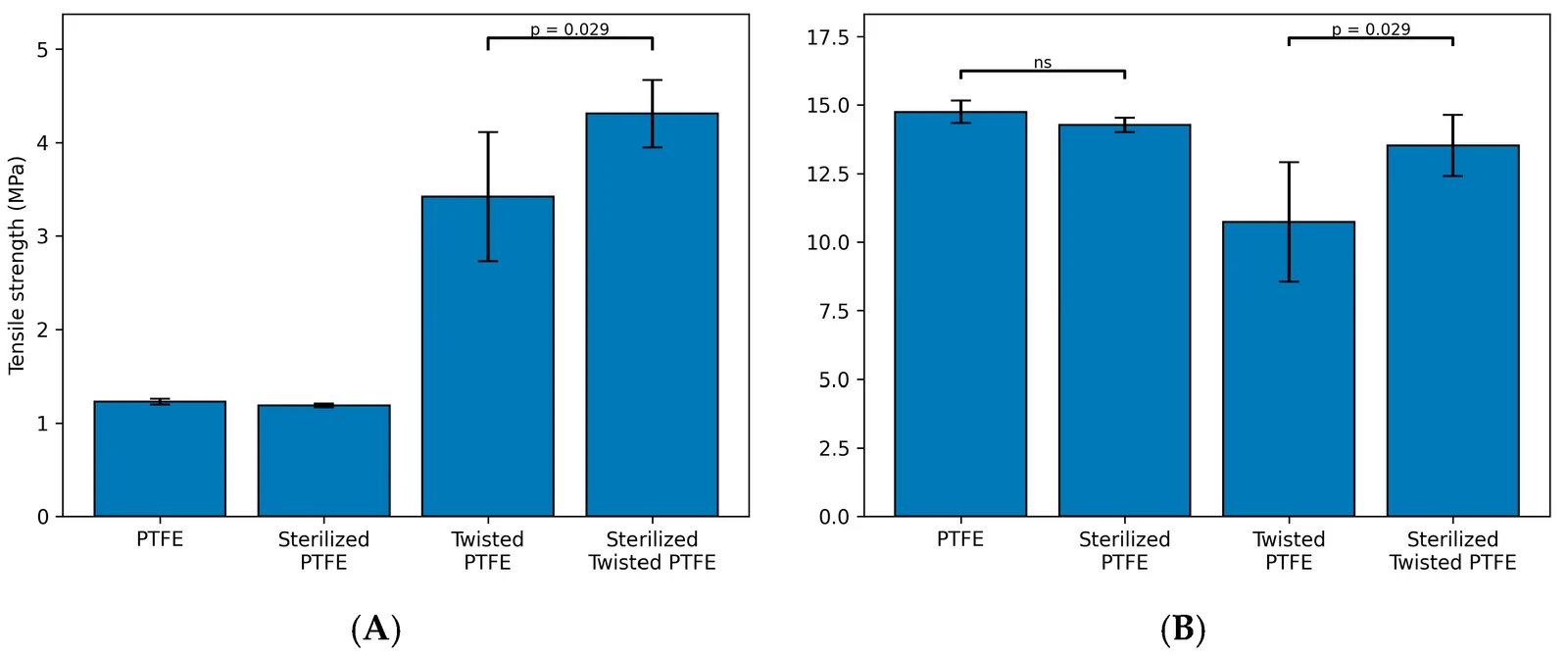
Mechanical properties of PTFE tape specimens: (**A**) Mean tensile strength values of non-sterilized PTFE, sterilized PTFE, twisted PTFE, and sterilized twisted PTFE specimens. (**B**) Mean maximum load at failure recorded during tensile testing. Data are presented as mean ± SD (*n* = 8). Error bars represent the standard deviation. Sterilized twisted PTFE exhibited significantly higher tensile strength and maximum load than twisted PTFE (*p* = 0.029), whereas no significant differences were observed between non-sterilized and sterilized PTFE specimens (*p* = 0.061). ns: no significant.

The tensile properties of PTFE tape were evaluated under four experimental conditions: non-sterilized PTFE, sterilized PTFE, twisted PTFE, and sterilized twisted PTFE (Figure 4).

For the non-sterilized PTFE tape, the mean tensile strength was 1.23 ± 0.03 MPa, while the maximum load sustained before failure reached 14.75 ± 0.41 N. Sterilization resulted in a slight reduction in both tensile strength (1.19 ± 0.02 MPa) and maximum load (14.28 ± 0.26 N). However, the differences between sterilized and non-sterilized specimens were not statistically significant (*p* = 0.061).

A substantial increase in tensile strength was observed after twisting the PTFE tape. The twisted PTFE specimens exhibited an average tensile strength of 3.42 ± 0.69 MPa, representing an increase of approximately 178% compared with the non-twisted specimens. The corresponding maximum load at failure was 10.74 ± 2.18 N.

The sterilized twisted specimens demonstrated the highest mechanical performance among all investigated groups, with a mean tensile strength of 4.31 ± 0.36 MPa and a maximum load of 13.53 ± 1.12 N. Compared with the twisted non-sterilized specimens, sterilization of the twisted tape produced a statistically significant increase in mechanical resistance (*p* = 0.029).

The results indicate that sterilization alone did not significantly affect the tensile properties of PTFE tape. In contrast, twisting the material markedly enhanced its tensile strength, while the combination of twisting and sterilization yielded the highest strength values recorded during the study.

### 3.3. SEM Evaluation

#### 3.3.1. SEM Analysis of Sterilized and Non-Sterilized PTFE Tape

**Figure 5:**
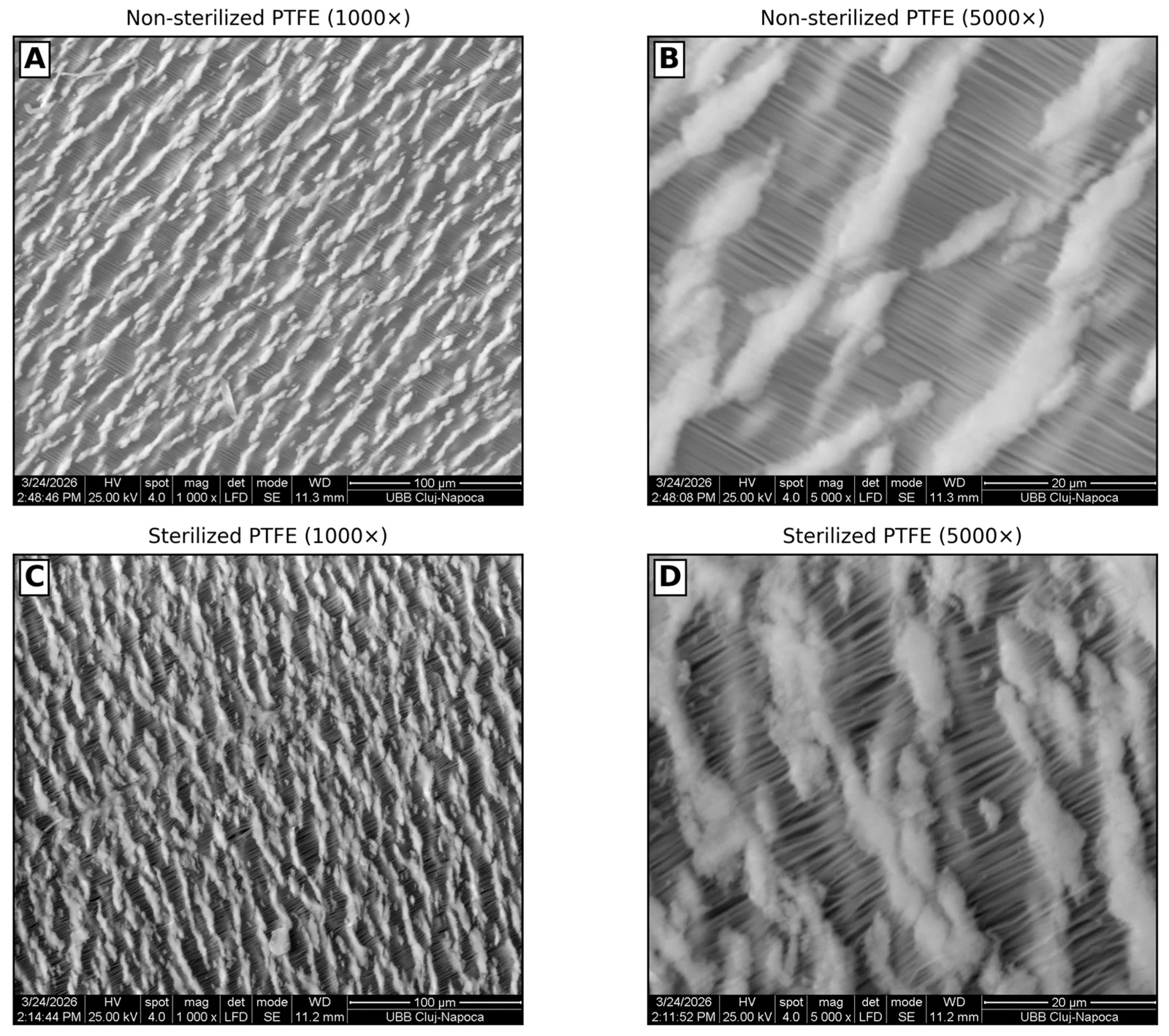
SEM micrographs of PTFE tape before and after sterilization: (**A**) Non-sterilized PTFE tape at 1000× magnification. (**B**) Non-sterilized PTFE tape at 5000× magnification. (**C**) Sterilized PTFE tape at 1000× magnification. (**D**) Sterilized PTFE tape at 5000× magnification. Non-sterilized PTFE tape at 1000× and 5000× magnification showed a fibrillar surface morphology with elongated longitudinal structures. Sterilized PTFE specimens displayed a comparable fibrillar architecture, with no evident signs of surface degradation, melting, cracking, or structural collapse.

SEM examination revealed that both sterilized and non-sterilized PTFE tape specimens exhibited a characteristic fibrillar surface morphology. At 1000× magnification, the non-sterilized PTFE tape showed an organized surface pattern composed of elongated, parallel fibrillar structures and irregular superficial ridges distributed along the longitudinal direction of the material. A similar morphology was observed in the sterilized specimens, with no evident disruption of the fibrillar arrangement (Figure 5A,C).

At higher magnification (5000×), both groups displayed fine, closely packed fibrillar elements and elongated surface formations. The non-sterilized specimens presented a continuous fibrillar network with localized irregularities, while the sterilized PTFE tape maintained a comparable microstructural organization. No clear evidence of thermal degradation, melting, extensive cracking, fibril rupture, or collapse of the surface architecture was observed after sterilization (Figure 5B,D).

Overall, SEM analysis indicated that sterilization did not induce relevant morphological alterations in the PTFE tape surface under the conditions of the present study.

#### 3.3.2. SEM Analysis of the Interaction Between Gingival Displacement Materials and Impression Material

SEM examination revealed distinct differences in the surface morphology of the addition silicone impression material following removal of the two gingival displacement materials.

**Figure 6:**
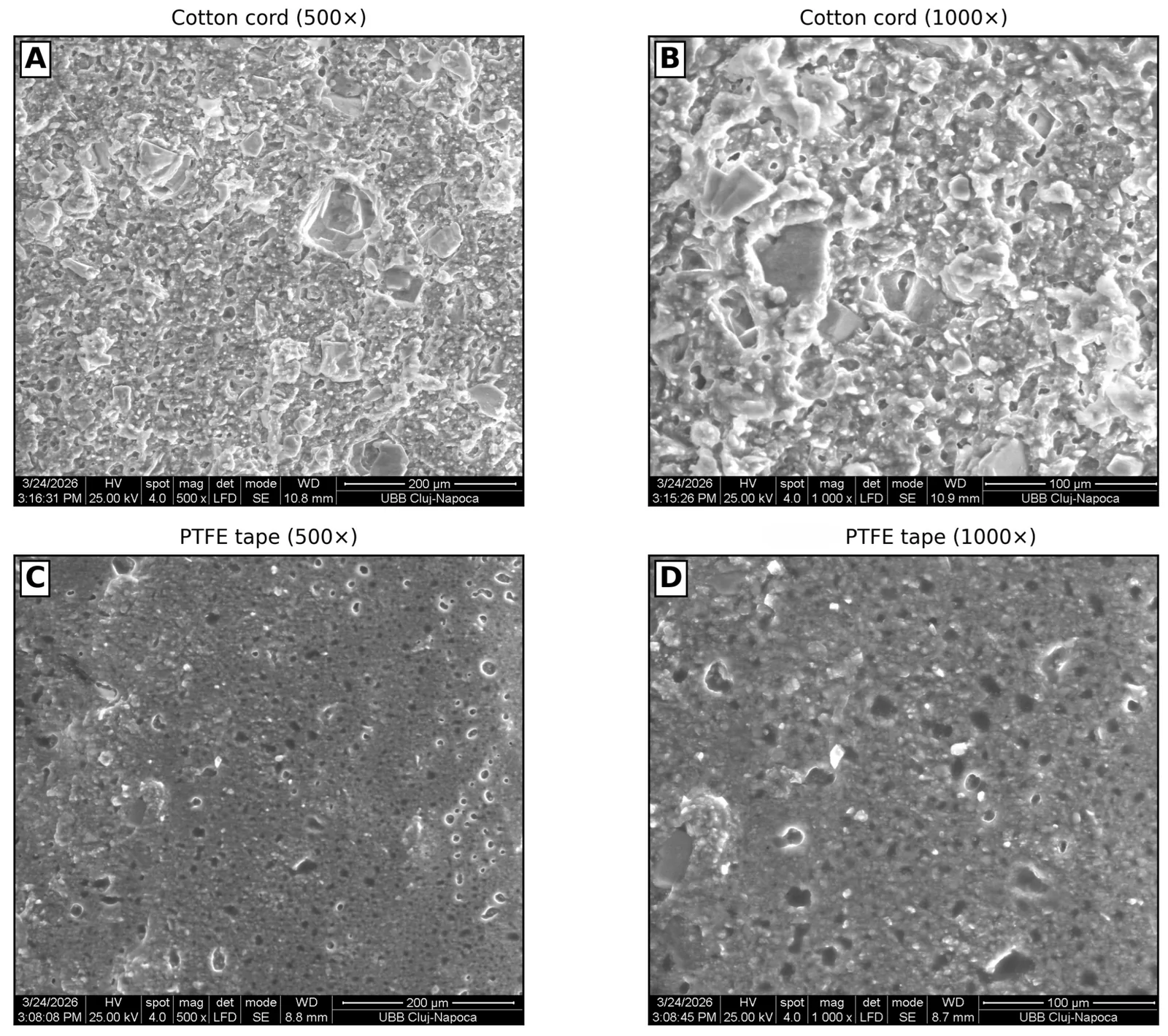
SEM analysis of the addition silicone impression surface following removal of gingival displacement materials: (**A**) Cotton retraction cord at 500× magnification. (**B**) Cotton retraction cord at 1000× magnification. (**C**) PTFE tape at 500× magnification. (**D**) PTFE tape at 1000× magnification. (**A,B**) Impression surface obtained after removal of the cotton retraction cord (500× and 1000×), showing irregular depressions, microporosities, and heterogeneous surface features. (**C,D**) Impression surface obtained after removal of PTFE tape (500× and 1000×), showing a qualitatively more homogeneous surface morphology based on SEM observations.

The specimens obtained after removal of the cotton retraction cord exhibited a heterogeneous surface characterized by irregular depressions, localized voids, and discontinuous surface features. At both 500× and 1000× magnifications, numerous surface irregularities and microporosities were observed, indicating a complex interaction between the fibrous structure of the cotton cord and the impression material during polymerization and removal (Figure 6A,B).

In contrast, the specimens associated with PTFE tape displayed a more homogeneous and continuous surface morphology based on qualitative SEM observations. The impression surfaces appeared to exhibit well-defined linear imprints with fewer visible discontinuities than those observed in the cotton retraction cord group. At higher magnification, the PTFE group showed a relatively smooth and uniform topography, with limited visible evidence of material disruption or surface damage (Figure 6C,D).

Overall, qualitative SEM observations indicated differences in the surface morphology of the impression material following removal of the two gingival displacement materials. Compared with the cotton retraction cord group, the PTFE group appeared to exhibit a more homogeneous surface morphology with fewer visible irregularities. Because the SEM evaluation was qualitative, these observations should be interpreted descriptively and do not constitute quantitative evidence of improved surface quality. The observed morphological differences may be related to the non-fibrous and non-adhesive nature of PTFE tape, although this hypothesis requires confirmation through quantitative surface characterization.

## 4. Discussion

The present in vitro study evaluated the effectiveness of polytetrafluoroethylene tape as an alternative gingival displacement material by combining dimensional CAD/CAM analysis, mechanical characterization, and scanning electron microscopy investigations. The null hypothesis was rejected, as statistically significant differences in gingival sulcus enlargement were observed among the experimental conditions. In addition, qualitative differences were identified in the interaction between the displacement materials and the impression material.

The primary finding of the study was that PTFE tape produced the highest mean sulcular enlargement (0.907 ± 0.099 mm), followed by the conventional cotton retraction cord (0.851 ± 0.140 mm) and the control condition (0.716 ± 0.118 mm). Repeated-measures ANOVA demonstrated a significant effect of the gingival displacement condition on sulcular width, and post hoc analysis showed that both displacement materials produced significantly greater sulcular enlargement than the control condition. Furthermore, PTFE tape achieved significantly greater sulcular enlargement than the conventional cotton retraction cord. These findings indicate that PTFE tape provides effective gingival displacement under standardized laboratory conditions. This observation is consistent with previous clinical investigations reporting favorable gingival displacement obtained with PTFE tape. Nasim et al. reported that PTFE tape provided gingival displacement and bleeding control comparable to conventional retraction cords while offering favorable handling characteristics [33]. Similarly, Ruggiero et al. demonstrated that PTFE can be successfully used for gingival displacement during intraoral scanning procedures, suggesting its potential as an alternative to conventional materials [34].

The greater gingival displacement achieved with PTFE tape in the present study may be attributed to its ability to closely adapt to the gingival sulcus and exert controlled lateral pressure on the surrounding soft tissues. Unlike conventional cotton retraction cords, which consist of intertwined fibers, PTFE tape can be compacted into the sulcus as a continuous material, allowing intimate adaptation to the sulcular anatomy and potentially promoting a more homogeneous distribution of displacement forces along the finish line. These findings are consistent with the observations of Castelo-Baz et al., who reported that the excellent adaptability, ease of manipulation, and effective condensation of PTFE tape within the gingival sulcus make it a suitable alternative for gingival displacement while minimizing trauma to the surrounding tissues [35]. Although the difference between PTFE tape and cotton retraction cord reached statistical significance under the standardized conditions of the present in vitro study, its clinical relevance should be interpreted with caution and requires confirmation in well-designed clinical investigations.

SEM analysis of the impression surfaces provided additional insight into the interaction between the displacement materials and the addition silicone impression material. The specimens associated with cotton retraction cord exhibited a more heterogeneous surface characterized by depressions, microporosities, and irregular surface features. In contrast, the PTFE specimens displayed a qualitatively more homogeneous and continuous morphology with fewer apparent defects. These observations may be explained by the intrinsic physicochemical properties of PTFE, including its hydrophobicity, chemical inertness, and non-fibrous structure. Unlike cotton fibers, which may mechanically interact with the impression material during polymerization and removal, PTFE may exhibit less mechanical interaction with the silicone matrix during removal. Although the SEM findings were qualitative, they suggest morphological differences in the interaction between the displacement materials and the impression material. Quantitative surface characterization is required to determine whether these observations translate into differences in impression surface quality or clinical impression accuracy.

The mechanical characterization of PTFE tape demonstrated that sterilization alone did not significantly affect tensile strength or maximum load values. SEM examination further confirmed the preservation of the characteristic fibrillar microstructure of expanded PTFE following sterilization. No evidence of fibril rupture, melting phenomena, microcracking, or structural collapse was identified. These findings are particularly relevant from a clinical perspective, as sterilization is often required before intraoral use. The ability of PTFE tape to maintain both structural integrity and mechanical performance after sterilization supports its potential use in clinical gingival displacement procedures.

An additional finding of the present study was the marked increase in tensile strength observed after twisting the PTFE tape. Twisted specimens exhibited substantially higher tensile strength values than non-twisted specimens, while sterilized twisted PTFE demonstrated the highest resistance among all investigated groups. Although tensile resistance is not the primary determinant of gingival displacement effectiveness, improved mechanical stability may facilitate handling and insertion of the material into a narrow gingival sulcus without fragmentation or deformation.

From a clinical standpoint, PTFE tape offers several potential advantages. The material is inexpensive, readily available, chemically inert, easily sterilized, and simple to manipulate. In addition, previous studies have reported lower microbial contamination of PTFE compared with cotton spacer materials. Although these findings cannot be directly extrapolated to gingival displacement procedures, they suggest that the hydrophobic and non-porous surface of PTFE may reduce bacterial retention compared with fibrous cotton materials [36,37,38,39]. Furthermore, the absence of hemostatic or vasoconstrictive agents eliminates the risk of chemical interactions with impression materials and reduces the likelihood of tissue irritation associated with medicated retraction cords. In the present study, PTFE tape produced significantly greater gingival sulcular enlargement than the conventional cotton retraction cord under standardized laboratory conditions and was associated with a qualitatively more homogeneous impression surface morphology. Nevertheless, the clinical significance of these findings should be interpreted with caution until confirmed by well-designed in vivo studies.

Several limitations should be considered when interpreting the present findings. The study was performed using a standardized three-dimensional printed model with artificial gingival tissues and, therefore, could not fully reproduce the anatomical dimensions, biomechanical behavior, and biological characteristics of the natural gingival sulcus and surrounding soft tissues. Although this experimental model provided reproducible conditions for direct comparison of the tested gingival displacement materials, the findings should be regarded as preliminary laboratory evidence and cannot be directly extrapolated to clinical performance in vivo. Factors such as gingival crevicular fluid, bleeding, tissue inflammation, patient-related variability, and operator-dependent clinical procedures were not represented in the experimental setup. In addition, the SEM observations were qualitative and did not include quantitative surface roughness measurements. Future studies should incorporate well-designed clinical investigations together with quantitative surface analyses to further evaluate the clinical performance of PTFE tape, its influence on impression accuracy, and its effects on periodontal tissues.

Another limitation of the present study is that the artificial gingival model does not fully reproduce the dimensions and biomechanical behavior of the natural gingival sulcus. Furthermore, the mechanical properties of the artificial gingival material were not quantitatively characterized. The same commercially available silicone material was used for all specimens to provide standardized and reproducible experimental conditions for comparing the evaluated gingival displacement materials. Consequently, the absolute sulcular width values recorded in the present investigation should not be interpreted as physiological measurements but rather as standardized laboratory measurements that allow direct comparison of the evaluated displacement materials.

Although all gingival displacement procedures were performed by the same operator using the same packing instrument and standardized insertion technique, the insertion force was not quantitatively measured. Therefore, slight variations in packing pressure cannot be completely excluded and should be considered when interpreting the findings. Future studies should incorporate objective measurements of insertion force to further improve methodological standardization.

The relatively small sample size represents an additional limitation of the present study. Although the repeated-measures design increased the efficiency of the statistical analysis, no a priori sample size or power calculation was performed, as the study was designed as a preliminary in vitro investigation. Therefore, further studies including larger sample sizes are required to confirm the present findings.

The present study demonstrated that PTFE tape produced the highest mean gingival sulcular enlargement among the evaluated conditions and significantly greater sulcular enlargement than the conventional cotton retraction cord within the standardized laboratory model. In addition, the material maintained its microstructural integrity following sterilization, exhibited favorable mechanical properties, and was associated with a qualitatively more homogeneous impression surface morphology based on SEM observations. Taken together, these findings support the potential of PTFE tape as an alternative gingival displacement material for fixed prosthodontic procedures. However, confirmation through adequately powered clinical studies is required before these laboratory findings can be translated into routine clinical practice.

## 5. Conclusions

Within the limitations of this standardized in vitro model, PTFE tape produced the highest mean gingival sulcular enlargement among the evaluated conditions and demonstrated effective gingival displacement under the tested experimental conditions. Under the tested sterilization conditions, no obvious microstructural deterioration was observed, and the mechanical properties of non-twisted PTFE were not significantly affected. The SEM observations of the material–impression interface were exploratory and qualitative and should therefore be interpreted with caution. Further replicated laboratory studies and well-designed clinical investigations are required before conclusions can be drawn regarding the clinical effectiveness, periodontal safety, hemostatic performance, and patient-related outcomes of PTFE tape.

## Data Availability

The original contributions presented in this study are included in the article. Further inquiries can be directed to the corresponding authors.

## Abbreviations

The following abbreviations are used in this manuscript:

PTFE	Polytetrafluoroethylene
CAD/CAM	Computer-Aided Design/Computer-Aided Manufacturing
SEM	Scanning electron microscopy
GD	Gingival displacement
3D	Three-dimensional
SD	Standard deviation
